# Videocapillaroscopic Alterations in Alopecia Areata

**DOI:** 10.1155/2013/160203

**Published:** 2013-09-19

**Authors:** Agnieszka Gerkowicz, Dorota Krasowska, Aldona Pietrzak, Anna Michalak-Stoma, Joanna Bartosińska, Maria Juszkiewicz-Borowiec, Grażyna Chodorowska

**Affiliations:** Department of Dermatology, Venereology and Paediatric Dermatology, Medical University of Lublin, Radziwiłłowska 13, 20-080 Lublin, Poland

## Abstract

Alopecia areata (AA) is a common hair disorder observed in dermatological practice; however, the exact mechanisms that lead to the hair loss are still unknown. Disturbances in the blood supply of hair follicles may be one of the elements in the complex pathogenesis of AA. Nailfold videocapillaroscopy is a noninvasive technique that allows analysis of skin microcirculation in vivo. The aim of the study was the videocapillaroscopic assessment of skin microcirculation in AA patients. The study included 44 patients with patchy alopecia areata, 27 with alopecia universalis or totalis, and 40 healthy volunteers. Nailfold videocapillaroscopy was performed in all participants according to a standard protocol. Obtained images were assessed qualitatively and quantitatively. Two types of videocapillaroscopic images were distinguished in the study. Abnormal videocapillaroscopic images were found in 42% of patients. Tortuous and branching capillaries (*P* = 0.013, *P* = 0.001), decreased density of capillaries (*P* = 0.009), enlargement of the efferent limb (*P* < 0.017), or top part of the loop (*P* = 0.009) were observed significantly more often than in the control group. Only some patients with AA presented with microvascular abnormalities characterised by altered videocapillaroscopic images. More studies, including larger group of patients with AA, are required to determine the role of observed videocapillaroscopic alterations in AA.

## 1. Introduction

Alopecia areata (AA) is a disease of a hair-bearing area [1–3]. Its incidence varies among different authors, ranging from 0.1 to 3.8% [1, 4–6].

The sudden onset of well-defined oval or round, single or multiple patches of nonscarring hair loss is a typical feature of AA [1, 3, 4, 6, 7]. More extensive hair loss leads to total loss of scalp hair (alopecia totalis (AT)) or total loss of scalp and body hair (alopecia universalis (AU)) [1, 3, 5]. Other clinical features of AA are nail changes, found in 7–66% of patients [1, 8].

Nowadays, AA is regarded as a T cell-mediated autoimmune disease of hair follicles. Genetic and environmental factors are also considered in its pathogenesis [1, 9]. It is recognised that adequate blood supply of the hair follicle is crucial for normal hair growth [10] and angiogenesis is strongly associated with the hair cycle [11–13]. Therefore, it has been suggested that disturbances in the blood supply of hair follicles may be one of the elements in the complex nature of AA [14, 15]. It is thought that a proangiogenic factor such as VEGF (vascular endothelial growth factor) may also be engaged in the pathogenesis of this disease. It was histologically demonstrated that decreased VEGF synthesis is associated with reduced vascularisation of the affected skin in patients suffering from AA. Furthermore, an increase of VEGF in hair follicle keratinocytes after DPCP (diphenylcyclopropenone) therapy resulted in an increase in the number of capillary vessels [14]. 

Capillaroscopy is a noninvasive imaging technique that enables assessment of skin microcirculation in vivo [16]. Videocapillaroscopy is a modern variant of this technique which uses an optical fiber probe connected to a microvideotelecamera and allows morphologic and functional analysis of microcirculation in higher magnification (usually 200x) [16, 17]. Videocapillaroscopy is mainly performed at the nailfold where capillaries run parallel to the skin, which enables visualisation of the whole length of the capillary loops. It is assumed that any alterations in microcirculation should be noticeable in the nailfold and thus capillaroscopy may be performed in all clinical conditions in which microcirculation involvement is expected [16, 18].

 The aim of the study was the videocapillaroscopic assessment of skin microcirculation in patients with AA.

## 2. Subjects and Methods

71 patients with AA (33 females, 38 males), aged between 22 and 56 (mean age 40.02 ± 11.02 years), were enrolled in this study. The control group consisted of 40 healthy volunteers (19 females, 21 males), aged between 21 and 56 (mean age 40.89 ± 9.88 years). AA was diagnosed in all patients. 44 patients (62%) had patchy AA, and 27 (38%) had extensive hair loss including AT, AU, and AU/AT. In 27 patients, changes of the fingernail plates, including pitting, trachyonychia, and onychorrhexis (resp., in 14, 10, and 3 patients), were recognised.

The excluding criteria were ages <18 and >60; coexisting diseases such as connective tissue diseases, lung diseases, hyperthyreosis and hypothyreosis, hypertension, types I and II diabetes, lipid abnormalities, ischemic heart disease, chronic venous insufficiency, and acrocyanosis; any nail plate involvement that resulted from a clinical condition other than AA; nicotine addiction or alcohol abuse; or manicure within 3 weeks prior to the videocapillaroscopy.

In all patients and in the control group, the nailfold videocapillaroscopy was performed using VideoCap 3.0 (DS Medica, Italy), 200x magnification. Videocapillaroscopy was performed in the morning to maintain the same conditions of the examination. All participants refrained from eating, drinking caffeinated drinks, and taking any drugs that might influence the circulatory system. Before the examination, each subject was adapted to the room temperature of  20–23°C for around 15–20 minutes. Nailfolds of the 2nd to 5th fingers of both hands were examined after applying a drop of cedar oil on the nailfold to obtain best visibility of the capillary network. Photos of each examined finger were taken and stored. The parameters that were evaluated during videocapillaroscopy are shown in Table 1. 

Two-proportion *z*-test was used to compare the videocapillaroscopic pattern between patients with AA and the control group. Pearson's chi-squared test was used to test associations between categorical variables. The level of significance was *P* < 0.05. Data analysis was performed using STATISTICA 9.0 software and Microsoft Excel 2007. 

## 3. Results

Tortuous loops observed in 84.5% of patients with AA were the most common videocapillaroscopic findings in patients with AA. Among these patients, 57.75% had only single tortuous loops, while in 26.76% multiple tortuous loops were observed. The frequency of tortuosity was statistically higher in the studied group than in the control group (*P* = 0.013). Branching capillaries and features of neovascularisation were observed significantly more often in the patients with AA when compared to the control group (*P* = 0.001 and *P* = 0.021, resp.). The nailfold videocapillaroscopy showed enlargement of the top part of the loop in 12.68% of patients, while 11.27% of patients had dilatation of the efferent limb. The frequency of these alterations was significantly higher in the studied group than in the control group (*P* = 0.009 and *P* < 0.017, resp.). It is worth noting that a decreased density of capillaries was observed significantly more often in the patients with AA than in the control group (*P* = 0.009). None of the patients had extremely elongated loops. The results of all the videocapillaroscopic parameters investigated in both groups are shown in Table 2.

Based on the results, two types of videocapillaroscopic images in the patients with AA were distinguished. The first, normal pattern (or close to the one observed in healthy population) was shown in 41 (57.8%) patients. Among them, 23 people had patchy AA and 18 had extensive hair loss. The second, abnormal pattern was observed in 30 (42.2%) patients, including 21 people with patchy AA and 9 with extensive hair loss. There was no statistical correlation between the type of AA and the presence of abnormal videocapillaroscopic images (*P* = 0.233).

**Figure 1:**
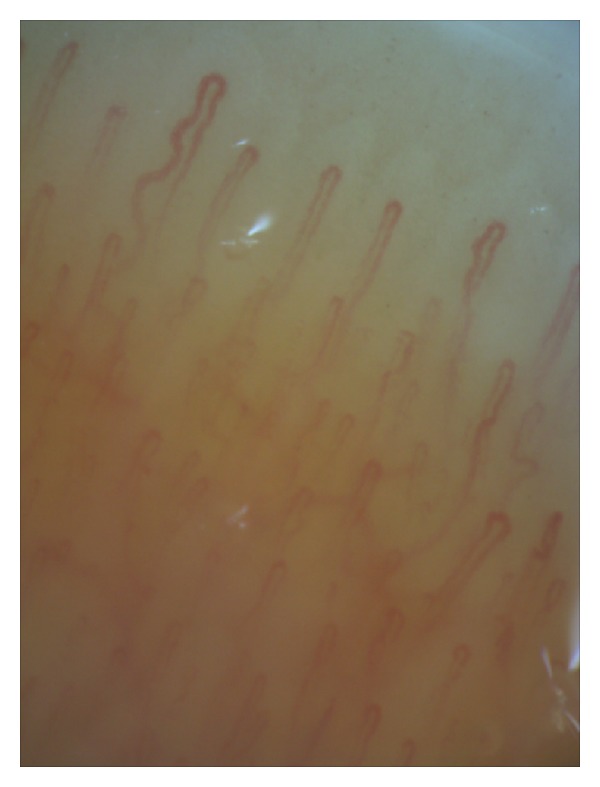
The first type of videocapillaroscopic images (normal): pink background, normal loop density, and loop size single tortuous capillaries (200x magnification).

In the first type, the capillaries had normal density, along with regular distribution and loop size. The background was pink. The majority of the loops had a characteristic appearance, similar to an inverted letter U; however, some patients had single tortuous loops with an irregular appearance. Subpapillary venular plexus was visible in some patients (Figure 1).

**Figure 2:**
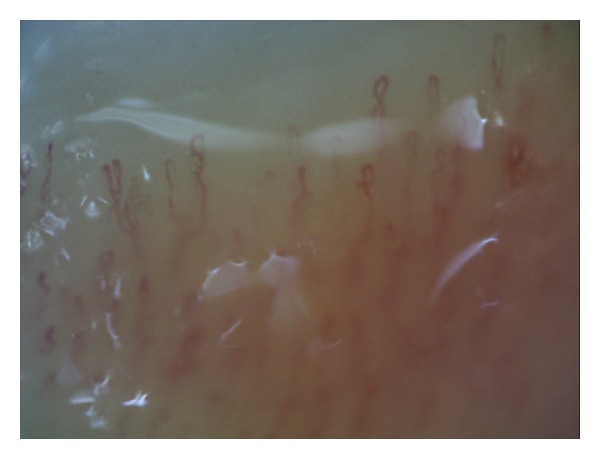
The second type of videocapillaroscopic images (abnormal): reduced density of capillaries, single tortuous loops, and branching capillaries (200x magnification).

**Figure 3:**
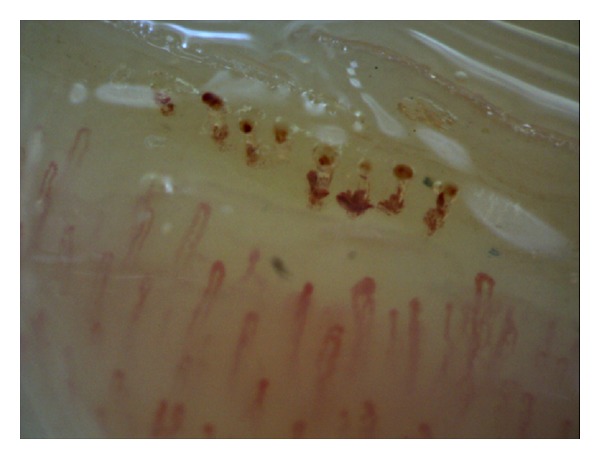
The second type of videocapillaroscopic images (abnormal): enlargement of the top part of the loop, features of capillary neoformation, and extravasations (200x magnification).

**Figure 4:**
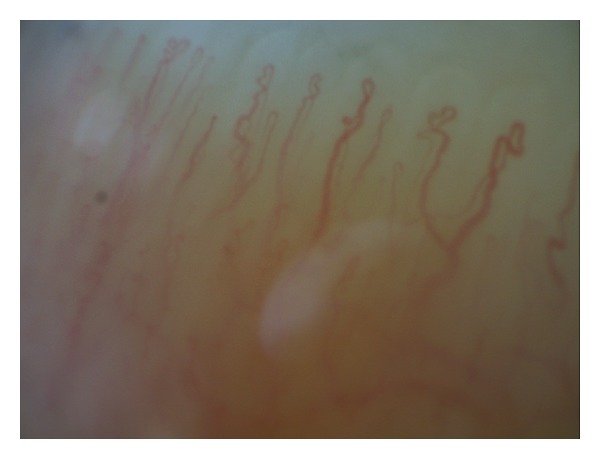
The second type of videocapillaroscopic images (abnormal): multiple tortuous loops with irregular appearance, dilatation of the efferent limb, and features of capillary neoformation (200x magnification).

In the second type, the videocapillaroscopic images were varied. However, each patient had at least 2 videocapillaroscopic alterations in minimum 2 fingers, such as multiple tortuous loops, branching capillaries, features of capillary neoformation characterized by thin branching within one limb or thin connection between the afferent and efferent limb, increased or decreased density of the capillaries, enlargement of the loop size, or altered distribution of the capillary network (Figures 2, 3, and 4). 

Among patients with the second type of videocapillaroscopic pattern, 8 (30.77%) patients had nail abnormalities and 22 (48.89%) did not have any nail changes. Moreover, we observed no statistical correlation between the occurrence of the second type of videocapillaroscopic pattern and the presence of nail changes (*P* = 0.137). 

## 4. Discussion

AA is a common hair disorder observed in dermatological practice; however, the exact mechanisms that lead to the hair loss are still unknown [1, 2, 6, 19, 20]. The possible role of microvascular disturbances has been suggested in the pathogenesis of AA [10–15]. Nailfold vidoecapillaroscopy is regarded as the most appropriate noninvasive technique which allows direct observation of skin microcirculation in vivo [16–18]. There are only a few studies evaluating capillaroscopic abnormalities in patients with AA [21, 22]. To the best of our knowledge, nailfold videocapillaroscopy has never been used to observe the microcirculation characteristics of alopecia areata.

In the current study, based on the videocapillaroscopic findings, two types of videocapillaroscopic images were distinguished in the patients with AA. 

57.8% of patients from the study group were classified as having the first type of videocapillaroscopic images. The findings including pink background, regular distribution of the capillary network, along with normal loop density and loop size were similar to the images observed in our control group as well as in the healthy population described in other studies [16]. Within this group, some patients had single tortuous capillaries. It is thought that the tortuosity of the loops may appear in the healthy population [16, 18]. According to some authors, the presence of more than 20% of the tortuous loops may be recognized as a characteristic for microangiopathy [16]. Therefore, in this study, only the presence of multiple tortuous loops was classified as abnormal. Neither branching capillaries nor the features of capillary neoformation were observed in this group.

 The second type of videocapillaroscopic pattern was identified in 42.2% of patients. The videocapillaroscopic findings varied among the patients, which may suggest different mechanisms that lead to the alteration in the microcirculation. 

All patients with the second type of videocapillaroscopic pattern had multiple or single tortuous loops, the majority of which had an irregular appearance. In three patients, “coiled” loops were observed. It is worth noting that in this group, in contrast to the healthy controls or to the patients with the first type of videocapillaroscopic pattern, the presence of branching capillaries and features of capillary neoformation were shown. These videocapillaroscopic findings may suggest angiogenesis which can be considered to be an attempt to compensate the loss of capillaries [18, 23]. It cannot be excluded that alterations in microcirculation observed in our study might appear in response to reduced density of capillaries. It is essential to highlight that a decreased density of capillaries was observed only in patients with the second type of videocapillaroscopic pattern. 

 Other alterations included enlargement of the top part of the loop or its efferent limb. None of the patients had giant capillaries. The enlargement of the diameter of capillaries was observed in 24% of patients, which is in accordance with data published by other authors. Rubisz-Brzezinska and Bendkowski [21] performed nailfold capillaroscopy examination in 34 patients with AA. Their most prominent findings were the enlargement of the top part of the capillary loop associated with single extravasations. The other most frequently observed alterations were homogenously enlarged loops which varied in number from single to multiple. The increase in number of enlarged capillary loops was associated with the occurrence of single extravasations [21]. In our study, enlargement of the top part of the capillary loop was also detected. Additionally, 26% of patients had extravasations, among them 12.68% had extravasations in at least three fingers, whereas the others had them in just one or two fingers. According to these findings, extravasations and enlargement of the top part of the loop may be considered as a characteristic feature of the capillaroscopic pattern in patients with AA. However, further studies, including a larger group of patients with AA, are required to confirm this observation.

 In contrast to the previous research, we did not observe homogenously enlarged loops. However, the enlargement of the efferent limb was found in 11.27% of patients with AA. It cannot be excluded that the differences resulted from a more precise diagnostic technique used in our study. 

It is worth to note that the enlargement of capillary loops is not characteristic for only one disease, because similar changes were found in diabetes types I and II [24], acrocyanosis, [25] or chronic autoimmune thyroiditis [26]. In our study all participants did not suffer from any of these diseases. Therefore these morphologic alterations need to be assessed in the context of the other videocapillaroscopic parameters.

Videocapillaroscopic images observed in our study indicate the variability of changes in patients with AA. It is considered that in autoimmune diseases the variability of morphological alterations observed during capillaroscopic examinations may result from a different intensity of the pathological changes in the vascular bed [27]. 

We did not find any significant correlation between the presence of abnormal videocapillaroscopic images and the type of AA. However, the presence of videocapillaroscopic alterations in some patients with localised AA as well as in some patients with more extensive hair loss may suggest the possible involvement of microvascular abnormalities in the course of alopecia areata. Further studies are required to confirm or refuse this hypothesis.

Apart from hair abnormalities in the course of AA, nail abnormalities are also observed [1, 8]. It could be expected that disturbances in the skin microcirculation may lead to the development of nail changes [28]. Surprisingly, abnormal videocapillaroscopic images were not prevalent in patients with nail changes. Moreover, they were observed only in 8 patients with nail involvement and in 22 patients with unaffected nails. Contrary to our results, Rubisz-Brzezinska and Bendkowski observed more frequently altered capillaroscopic images in patients with AA and with nail changes [21]. Due to the lack of other studies considering nailfold videocapillaroscopy in alopecia areata, the obtained results are difficult to interpret. 

Our study does not reveal specific videocapillaroscopic pattern in alopecia areata. We observed videocapillaroscopic alterations in 42.2% of patients. It may suggest some disturbances in microcirculation. It cannot be also excluded that more prominent changes will be observed within bald patches. Ganzetti et al. [22] performed videocapillaroscopic examinations of the scalp in patients with AA before and after the DPCP (diphenylcyclopropenone) therapy. In the study, the authors observed a significant increase of new vessels and a consequent hair regrowth in those places where features of neoangiogenesis were more marked after DPCP [22]. Therefore, it seems worth to compare videocapillaroscopic patterns of the nailfold with videocapillaroscopic images of the bold area in patients with AA.

## 5. Conclusions

Our results show that only some patients with AA presented abnormal videocapillaroscopic images. The videocapillaroscopic alterations observed in this group of patients may suggest disturbances in skin microcirculation in the course of alopecia areata. More studies including a larger group of patients with AA are required to determine the role of observed videocapillaroscopic alterations in alopecia areata. 

## Figures and Tables

**Table 1:** Videocapillaroscopic criteria.

Videocapillaroscopic parameters	Criteria
Distribution of the capillaries	
Regular	Homogenous distribution of capillaries
Altered	Nonhomogenous distribution of capillaries
Density of the capillaries	
Normal	9–13 capillaries/mm
Increased	>13 capillaries/mm
Decreased	<9 capillaries/mm or avascular area
Diameter of the capillaries	
Normal	The diameter of the afferent limb (6 to 19 *μ*m) and the diameter of the efferent limb (8–20 *μ*m), venous limb width ratio: arterial limb no greater than 2 : 1
Enlargement of the top part of the loop	Diameter of the limbs larger than average
Enlargement of the efferent limb	Diameter of the limbs larger than average
Length of the capillaries	
Normal	Approximately 400 *μ*m
Elongated	>750 *μ*m
Extravasation	
Absent	No extravasations
Single	Present in 1-2 fingers
Multiple	Present in >2 fingers
Subpapillary venular plexus visibility	
Invisible	
1-2 fingers	Visible in 1-2 fingers
>2 fingers	Visible in >2 fingers
Background colour	
Pale	
Pink	
Reddish	
Morphology of the capillaries	
Normal	Appearance like an inverted letter U
Tortuous capillaries—regular appearance	Single crossovers
Tortuous capillaries—irregular appearance	Multiple crossovers or appearance like “treble clef” loop, “antler” loop, “trefoil” loop or meandering appearance with single/multiple crossovers
The number of tortuous capillaries	
Single	<5 at least in two fingers
Multiple	>5 at least in two fingers
Branching capillaries	
Present	At least one such loop in two fingers
Coiled capillaries	
Present	At least one such loop in two fingers
Features of the capillary neoformation	
Present	Thin, branching from one limb, or thin interconnection between the limbs (at least one such loop in two fingers)

**Table 2:** Videocapillaroscopic findings in patients with alopecia areata and in the control group.

Videocapillaroscopic parameters	Alopecia areata	Control group	*P*
Distribution of the capillaries			
Regular	55 (77.46%)	40 (100%)	0.001
Altered	16 (22.53%)	0	
Density of the capillaries			
Normal	58 (81.7%)	40	—
Increased	2 (02.28%)	0	—
Decreased	11 (15.49%)	0	0.009
Diameter of the capillaries			
Normal	54 (76%)	40	—
Enlargement of the top part of the loop	9 (12.68%)	0	0.009
Enlargement of the efferent limb	8 (11.27%)	0	0.017
Length of the capillaries			
Normal	71 (100%)	40	—
Extravasations			
Absent	52 (73.24%)	36 (90%)	0.018
Single	10 (14.08%)	4 (10%)	
Multiple	9 (12.68%)	0	
Subpapillary venular plexus visibility			
Invisible	59 (83.10%)	34 (85%)	0.571
1-2 fingers	11 (15.49%)	6 (15%)	
>2 fingers	1 (1.41%)	0	
Background colour			
Pale	8 (11.27%)	0	0.002
Pink	63 (88.73%)	40 (100%)	—
Tortuous capillaries			
Absent	11 (15.49%)	14 (35%)	0.013
Single	41 (57.75%)	26 (65%)	
Multiple	19 (26.76%)	0	
Tortuous capillaries irregular appearance			
Absent	44 (61.97%)	25 (62.50%)	0.802
Present	27 (38.03%)	15 (37.50%)	
Branching capillaries			
Absent	49 (69.01%)	40 (100%)	0.001
Present	22 (30.99%)	0	
Coiled capillaries			
Absent	68 (95.77%)	40 (100%)	0.187
Present	3 (4.23%)	0	
Features of the capillary neoformation			
Absent	45 (63.38%)	40 (100%)	—
Present	26 (36.62%)	0	0.021
